# Complete Sequence and Annotation of the Mycoplasma phocidae Strain 105^T^ Genome

**DOI:** 10.1128/MRA.01237-18

**Published:** 2018-10-11

**Authors:** Salvatore Frasca, Gerald F. Kutish, Dina L. Michaels, Daniel R. Brown

**Affiliations:** a Department of Comparative, Diagnostic and Population Medicine, College of Veterinary Medicine, University of Florida, Gainesville, Florida, USA; b Department of Pathobiology and Veterinary Science and Center of Excellence for Vaccine Research, University of Connecticut, Storrs, Connecticut, USA; c Department of Infectious Diseases and Immunology, College of Veterinary Medicine, University of Florida, Gainesville, Florida, USA; Georgia Institute of Technology

## Abstract

The genome of Mycoplasma phocidae strain 105^T^ was analyzed in order to improve our understanding of its role in epidemic marine mammal mortalities. It was found to encode a suite of immunosuppressors that may enable evasion of host defenses and modulate susceptibility to viral coinfections or their severity in seals.

## ANNOUNCEMENT

Mycoplasma phocidae strain 105^T^ was first isolated from the lungs of one of more than 400 harbor seals (Phoca vitulina) that died along the U.S. New England coastline during a mass mortality event in 1980. Epidemic seal deaths occurring in the North Atlantic in 1988 and 2002 were attributed primarily to phocine distemper virus or H7N7 influenza virus, but coinfection with M. phocidae was common (1–3). In order to improve our ability to explain the role of M. phocidae in marine mammal mortalities and the implications of such events for human and ocean health, we sequenced and annotated the genome of a lyophilized specimen of strain 105^T^ acquired by the Mollicutes Collection (World Data Centre for Microorganisms Collection 858) from the ATCC (catalog number 33657) in 1990. The DNA was extracted using an Easy-DNA genomic DNA purification kit (catalog number K180001; Thermo Fisher Scientific). Sequencing libraries were constructed using the NEBNext UltraTMII DNA library prep kit (catalog number E7645S; New England Biolabs) and dual-index NEBNext Multiplex Oligos (catalog number E7600S; New England Biolabs). Illumina MiSeq 2 × 300-bp paired-end sequencing yielded approximately 5 × 10^7^ Phred quality-filtered reads with a quality score greater than 30. The final assembly with coverage depth of more than 800× was achieved in May 2018 by using a combination of Ray v2.3.1, Unicycler v0.4.3, and Consed v29 finishing software (4–6) and then annotated with the RAST server and the NCBI Prokaryotic Genome Annotation Pipeline (7, 8), all with default settings.

The closed circular genome is 814,478 bp long, with a G+C content of 27.1 mol%, close to the 27.8 mol% estimated by buoyant density (3). Of 657 predicted open reading frames (ORFs), about two-thirds have assigned functions, and about half could be grouped in subsystems, including 40 structural RNAs, 181 proteins involved in nucleic acid metabolism, transcription, translation, or protein fate, 45 in amino acid, purine, pyrimidine, nucleoside, nucleotide, cofactor, prosthetic group, or carrier metabolism, 27 in carbohydrate metabolism, 9 in fatty acid or phospholipid metabolism, 11 in transport or binding processes, 5 in cell division, and 8 chaperones, elongation factors, or transcription regulators. A complete arginine dihydrolase pathway of ATP synthesis is present as expected for this member of the Mycoplasma hominis phylogenetic cluster (9). We interpret the presence of genes encoding cytosine 5-methyltransferase (CpG DNA methylase), adenine N-6 methyltransferase, and type II and III restriction-modification system DNA methylases as evidence of the capacity for epigenetic modifications of the genome.

M. phocidae encodes several candidate virulence factors, including a family of putative surface antigens with about 60% amino acid sequence similarity over their proline-rich N-terminal region, to the pneumococcal complement evasion protein PspA (10); a family of proteins with about 40% sequence similarity to mycoplasmal protein M, which binds the light chain region of IgG to block antigen-specific binding (11); and a predicted immunoglobulin heavy chain-binding protein similar to the phocine host-specific IgG endoprotease IdeP of Streptococcus phocae (12) fused to the mycoplasmal lipoprotein LppA. It remains to be confirmed whether this suite of immunosuppressors constitutes a mechanism by which M. phocidae evades host defenses and predisposes susceptibility or modulates outcomes of influenza or distemper in seals.

### Data availability.

The M. phocidae 105^T^ genome sequence and annotation data have been deposited in GenBank under the accession number CP029295; the sequence described in this paper is the first version, CP029295.1. The raw data are available in the NCBI Sequence Read Archive under accession number SRX4702894.
